# Testing and Iterative Improvement of the CEN ISO/TS 82304-2 Health App Quality Assessment: Pilot Interrater Reliability Study

**DOI:** 10.2196/64565

**Published:** 2025-03-10

**Authors:** Anna-Lena Frey, Diana Matei, Ben Phillips, Adam McCabe, Rachel Fuller, Begoña Laibarra, Laura Alonso, Victor de la Hoz, Carme Pratdepadua Bufill, Berta Llebot Casajuana, Giuseppe D'Avenio, Pier Angelo Sottile, Laura Melania Rocchi, Matteo Errera, Yasmine Laaissaoui, Michael Cardinal, Menno Kok, Petra Hoogendoorn

**Affiliations:** 1 Organisation for the Review of Care and Health Apps (ORCHA) Daresbury United Kingdom; 2 Software Quality Systems Getxo Spain; 3 TIC Social Health Foundation Ministry of Health Government of Catalonia Barcelona Spain; 4 National Center for Innovative Technologies in Public Health National Institute of Health Rome Italy; 5 BI Health Srl Rome Italy; 6 BaasBox Srl Rome Italy; 7 TherAppX Granby, QC Canada; 8 EIT Health Belgium and The Netherlands Rotterdam The Netherlands; 9 National eHealth Living Lab Department of Public Health and Primary Care Leiden University Medical Center Leiden The Netherlands

**Keywords:** health apps, mobile health, digital health, quality evaluation, assessment framework, interrater reliability

## Abstract

**Background:**

With the increasing use of health apps and ongoing concerns regarding their safety, effectiveness, and data privacy, numerous health app quality assessment frameworks have emerged. However, assessment initiatives experience difficulties scaling, and there is currently no comprehensive, consistent, internationally recognized assessment framework. Therefore, health apps often need to undergo several quality evaluations to enter different markets, leading to duplication of work. The CEN ISO/TS 82304‑2 health app assessment seeks to address this issue, aiming to provide an internationally accepted quality evaluation through a network of assessment organizations located in different countries.

**Objective:**

This study aimed to develop and evolve the draft CEN ISO/TS 82304-2 assessment handbook and developer guidance by testing them across organizations in several countries.

**Methods:**

Assessment organizations from 5 countries were engaged to evaluate 24 health apps using the evolving CEN ISO/TS 82304-2 assessment across 3 evaluation rounds. The information submitted by a given health app developer was evaluated by 2 assessment organizations, and interrater reliability was examined. In addition, app developers and assessors were asked to report how much time they spent on information collation or evaluation and to rate the clarity of the developer guidance or assessor handbook, respectively. The collected data were used to iteratively improve the handbook and guidance between rounds.

**Results:**

The interrater reliability between assessment organizations improved from round 1 to round 2 and stayed relatively stable between rounds 2 and 3, with 80% (55/69) of assessment questions demonstrating moderate or better (Gwet AC1>0.41) agreement in round 3. The median time required by developers to prepare the assessment information was 8 hours and 59 minutes (IQR 5.7-27.1 hours) in round 3, whereas assessors reported a median evaluation time of 8 hours and 46 minutes (IQR 7.1-11.0 hours). The draft guidance and handbook were generally perceived as clear, with a median round-3 clarity rating of 1.73 (IQR 1.64-1.90) for developers and 1.78 (IQR 1.71-1.89) for assessors (0=“very unclear”, 1=“somewhat unclear”, and 2=“completely clear”).

**Conclusions:**

To our knowledge, this is the first study to examine the consistency of health app evaluations across organizations located in different countries. Given that the CEN ISO/TS 82304-2 guidance and handbook are still under development, the interrater reliability findings observed at this early stage are promising, and this study provided valuable information for further refinement of the assessment. This study marks an important first step toward establishing the CEN ISO/TS 82304-2 assessment as a consistent, cross-national health app evaluation. It is envisioned that the assessment will ultimately help avoid duplication of work, prevent inequities by facilitating access to smaller markets for developers, and build trust among users, thereby increasing the adoption of high-quality health apps.

## Introduction

### Background

Health apps, which include both medical and wellness apps, have the potential to greatly benefit patients, the public, health care professionals, and the broader health care system [1,2]. They can increase the effectiveness of health care services by supporting behavior change and improving clinical outcomes, enhance efficiency by preventing hospitalization and freeing up resources, and allow for remote monitoring and treatment and, as a result, can have a large positive socioeconomic impact [1,2].

However, alongside these potential benefits, there are several risks associated with the use of low-quality health apps. Previous research has found that many health apps demonstrate data privacy or security risks [3-6], raise clinical safety concerns [7], provide incorrect medical information [8,9], or are not supported by efficacy or effectiveness evidence [4,5,10].

With >350,000 health apps on the market [11], it is difficult for health care professionals and the public to distinguish between beneficial and potentially harmful or ineffective apps. This issue is exacerbated by the fact that readily available metrics such as user ratings and download numbers are not correlated with health app quality [12]. Moreover, concerns regarding effectiveness, safety, usability, and data privacy can be a barrier to the widespread adoption of health apps [13-15], thereby preventing the realization of their potential benefits.

Health app assessments can help address this issue by providing transparent quality information to the public, health care professionals, payers, medical societies, and health care organizations. However, there is currently no comprehensive and consistent assessment that is internationally recognized. While the certification provided under the European Union (EU) Medical Device Regulation (MDR) is valid across EU member states, a large proportion of health apps are not independently evaluated by notified bodies under the MDR. This is because many apps are not classified as medical devices or fall into risk class I, for which app developers only need to declare conformity based on a self-assessment that is not externally validated [16,17]. Furthermore, there are important aspects, such as data privacy, that are not usually independently examined as part of the MDR certification, and research shows that many health apps on the European market do not meet relevant data protection requirements [18]. Finally, the only output provided by the MDR assessment is a CE (European conformity) mark. Consequently, even for health apps that have been examined under the MDR, stakeholders need to carry out additional evaluations to gather more detailed quality information that is required to inform decision-making.

Partly as a result of these limitations of the MDR and similar regulations in other regions, a range of organizations have developed their own assessment frameworks to guide health app–related decision-making. This includes health authorities, charitable and academic institutions, patient and medical professional associations, and commercial companies [19,20]. The resulting fragmented assessment landscape leads to duplicated work for both health systems and app developers if a given app needs to undergo several assessments to evidence its quality for different markets and varying types of organizations.

The CEN ISO/TS 82304-2 health app assessment, which was developed with funding from the European Commission, seeks to address this issue. It aims to provide a comprehensive and consistent quality evaluation that is recognized across Europe and beyond without duplicating the legal tasks of notified bodies under the MDR [21,22].

In this context, a notable challenge is scaling the assessment to potentially cover a substantial proportion of the >350,000 health apps on the market [11]. Notably, a recent review reported that only a median of 45 evaluated digital health technologies, comprising mostly health apps, were available in the assessment libraries of 24 digital health assessment organizations [20]. This limited availability of evaluated apps is likely due to several factors. First, some assessments that require developers to actively submit their apps for evaluation may not offer sufficient incentives to lead to a substantial number of app submissions.For example, incentives may be insufficient if the assessments are not associated with a large enough increase in app users, are not integrated into health care system decision-making (eg, for procurement or reimbursement), or provide reimbursement payment conditions or postprescription procedures that are unappealing (eg, causing delays in patient access to the app) [23-27]. Second, app developers may not be able (due to limited funds and staff in start-ups and small companies) or willing (in light of lacking incentives) to invest the time and money required to meet the quality requirements of individual schemes, such as those requiring new clinical studies, which may affect the number of apps submitted to and passing particular assessments [26,28]. Third, difficulties in scaling the assessment process may play a role. A review focusing specifically on national health app evaluation initiatives found that even the front-runners in this area, such as Germany, Belgium, and the United Kingdom, are struggling with efficient assessment implementation. This may partly be due to the use of centralized approaches to app evaluation, which can create bottlenecks [29]. It has been proposed that international collaboration and the use of distributed evaluation bodies is needed to address this issue [29]. Following this approach, the CEN ISO/TS 82304-2 assessment is planned to be implemented across a network of assessment organizations in Europe and beyond to achieve efficient scaling while ensuring consistent assessment results across countries (and providing sufficient incentives, as explored in our recent discrete choice experiment [25]).

### Objectives

This study aimed to develop and evolve the CEN ISO/TS 82304-2 assessment handbook and developer guidance by testing the assessment across organizations located in different countries. For this purpose, assessment organizations from 5 countries were engaged to evaluate 24 diverse health apps using information submitted by the app developers. In total, 2 organizations assessed each app, and their interrater reliability was examined. In addition, app developers and assessors were asked to report how much time they spent collating or evaluating the assessment information, respectively, and to rate the clarity of the assessment instructions. The collected interrater reliability, time, and clarity rating data were used to iteratively improve the CEN ISO/TS 82304-2 assessment handbook and guidance across 3 evaluation rounds.

## Methods

### CEN ISO/TS 82304-2 Assessment Framework

The CEN ISO/TS 82304-2 assessment framework is intended to provide an independent, evidence-based, international evaluation of health app quality for a range of health care stakeholders. The framework was created using a Delphi methodology with input from 83 experts from 8 stakeholder groups, including medical organizations, health care authorities, and health app developers across 6 continents [21].

It should be noted that, while the CEN ISO/TS 82304-2 quality requirements are applicable worldwide, some requirements are influenced by local regulations (which is the case for other digital health assessment frameworks as well [29]). This necessitates minor amendments to the assessment across jurisdictions. Given that the project as part of which this study was conducted was funded by the European Commission, the initial CEN ISO/TS 82304-2 testing and implementation efforts were focused on Europe. Therefore, steps were taken to ensure that the CEN ISO/TS 82304-2 criteria are in line with European regulations and standards (as described further in the Discussion section). Future work will aim to support the alignment of the assessment with regulations in other regions.

The CEN ISO/TS 82304-2 framework consists of 5 subsections containing between 7 and 28 questions each: “Product information,” “Healthy and safe,” “Easy to use,” “Secure data,” and “Robust build.” In total, the assessment comprises 81 questions, 12 of which serve to collect background data about the app, mostly to enable the display of this information in a related health app quality label. As the latter questions are answered in free-text or multiple-selection format and do not affect the quality rating of the app, they were not considered in the interrater reliability analysis reported in this paper. The remaining 69 questions (quality requirements) have either 2 (“yes” or “no”) or 3 (“yes,” “no,” or “not applicable”) response options. A list of all assessment questions can be found in the supplement of the CEN ISO/TS 82304-2 framework development paper [21].

Final responses to the assessment questions are determined by an assessment organization based on documentation and other information that is provided by health app developers as part of the assessment process. Examples of the documentation that developers are asked to submit alongside providing access to their apps include evidence of the societal and health benefits of the app, an analysis of health risks, standard operating procedures (or relevant excerpts thereof), and privacy policies.

To facilitate the assessment process, detailed instructions were developed during this study to indicate what documentation is acceptable and what subrequirements need to be met for a given assessment question to be answered with “yes.” These instructions were outlined in a draft developer guidance document and a draft app assessor handbook and were tested and evolved across evaluation rounds (see the App Assessment Procedure section for details). As the handbook is still under development, it is not presented in this paper; however, it will likely be published once the first operational version is finalized.

The final output of the CEN ISO/TS 82304-2 assessment is a quality label [30] that summarizes the assessment results for each quality domain and for the app overall using letter grading (E to A) and color coding (red, orange, yellow, light green, and dark green), similar to the EU energy label. This allows health care professionals and the public to quickly determine whether a given app meets their needs and preferences. For more detailed information, a full assessment report is also available [22].

It should be noted that, as part of this study, no quality labels were issued given that the CEN ISO/TS 82304-2 assessment guidance and handbook were under development.

### App Selection

As mentioned previously, conducting the CEN ISO/TS 82304-2 assessment requires health app developers to submit documentation that is not publicly available. Therefore, developers were actively involved in this study, as is described further in the App Assessment Procedure section. This also allowed for the collection of feedback on the assessment process from the app developer perspective.

To thoroughly test the evolving assessor handbook, developer guidance, and assessment process, a variety of health apps were sought to be included in this study, mimicking real-life app diversity. For this purpose, the assessors involved in this study compiled a list of health app development companies to contact, aiming to cover a wide range of company sizes, countries of origin, condition areas, and app functionalities. In addition, this study was advertised on a dedicated project website where developers had the opportunity to get in touch to express their interest in participating.

To ensure that a given app could be evaluated by any assessor (see the App Assessment Procedure section for details), only apps available in English were eligible for inclusion in the study. However, in 2 cases, it was discovered, after the developers had collated all the assessment information, that their apps did not meet this criterion. Given that considerable effort had already been exerted at this stage, these apps were included, allocating them to assessors with relevant language knowledge or using automated translation tools where needed. No other eligibility criteria were applied apart from a willingness of the app developers to provide the necessary assessment information as well as access to their apps (or, as an exception, relevant screenshots or video demonstrations if access could not be granted, eg, due to geographical restrictions).

The assessment took place across 3 rounds, and the initial aim was to evaluate 8 health apps per round, with funding being available for the evaluation of 24 apps in total. However, due to developers’ competing commitments, the amount of effort involved, and the relatively short project timelines, a number of app development companies dropped out of the study after initially agreeing to take part. In round 1, timelines did not permit the recruitment of replacements. In subsequent rounds, more companies than needed were initially recruited to account for potential dropouts. The final sample consisted of the intended total of 24 health apps, which were distributed across the assessment rounds as follows: 3 (12%) apps in round 1 (3 dropouts), 7 (29%) apps in round 2 (7 dropouts and 1 moved to round 3), and 14 (58%) apps in round 3 (10 dropouts).

As mentioned previously, no quality labels were provided for the evaluated apps as part of this study given that the aim was to develop, test, and evolve the CEN ISO/TS 82304-2 assessment guidance and handbook to arrive at an operational version that is suitable for use in quality certification, with sufficiently consistent assessment results. Therefore, no letter grades were calculated or shared. The main benefits offered to participating health app development companies were the receipt of feedback for the individual assessment questions (based on the respective draft versions of the handbook), which could be used internally for product improvements; a chance to familiarize themselves with the CEN ISO/TS 82304-2 assessment; and the offer of a reduced fee if they decided to submit their apps to the operational version of the CEN ISO/TS 82304-2 certification once the latter is rolled out.

### App Assessment Procedure

The app evaluations were conducted between November 2022 and April 2024 by assessment organizations with diverse backgrounds from different countries to approximate the full scope of real-life assessment conditions. Specifically, participating organizations were located in the United Kingdom, Italy, Spain, Canada, and France and consisted of 3 private assessment organizations and 2 health authorities, the latter of which each used a subcontractor. In the final round, 1 assessment organization had to drop out as the assessors involved in the study had left the company. Other than that, the same organizations were involved in all 3 evaluation rounds.

Across all organizations, a total of 12 individual assessors were involved in the study, for 10 (83%) of whom background information was available (the remaining n=2, 17%, were based at the organization that had to drop out in the last round). The median previous experience that assessors had with evaluating the quality of health apps using any assessment framework was 19.5 (IQR 0.0-48.0) months. With regard to previous exposure to the CEN ISO/TS 82304-2 assessment in particular, 40% (4/10) of the assessors reported having previously used the CEN ISO/TS 82304-2 assessment to evaluate health apps, and 20% (2/10) of the assessors had been involved in the development of the framework. The remaining 40% (4/10) of the assessors had no previous familiarity with the CEN ISO/TS 82304-2 assessment.

The app assessment procedure across all 3 evaluation rounds was as follows. At the start of each round, the app developers who had agreed to participate in the study were supplied with the draft CEN ISO/TS 82304-2 assessment guidance for round 1, 2, or 3, as applicable. They were asked to collate and submit the required information, including relevant documentation, and grant the assessors access to their health app. In addition, developers were asked to report, for each question, how long it took them to gather the relevant information and how clear they found the associated assessment guidance (red=“very unclear,” yellow=“somewhat unclear,” and green=“completely clear,” coded as 0, 1, and 2, respectively, for the data analysis). Moreover, additional feedback was collected from app developers via individual interviews that were conducted via video calls after the submission of the assessment information but before the receipt of the evaluation results (see the Feedback From Developers section for details).

The assessment information submitted by a given app developer was passed on to 2 of the 5 assessment organizations, which were pseudorandomly allocated in such a way as to achieve diverse organization pairings across apps. A range of expertise was needed to complete the evaluations as the CEN ISO/TS 82304-2 assessment covers clinical, usability, privacy, and technical domains in depth. Therefore, some assessment organizations engaged different assessors to evaluate each of the assessment subsections, whereas others appointed a lead assessor who consulted subject matter experts when needed. The median number of apps evaluated by individual assessors across all 3 rounds with an evolving handbook was 9.5 (IQR 7.0-11.0).

As part of the app evaluation, assessors examined the submitted evidence, which included documentation and screenshots as well as relevant screens within the app itself based on the CEN ISO/TS 82304-2 draft handbook for round 1, 2, or 3 and recorded their responses (“yes,” “no,” or “not applicable”) for each question. Evaluations were conducted independently (ie, different assessment organizations did not have access to each other’s responses). Assessors also provided evaluation times and handbook clarity ratings for each of the 81 assessment questions.

After the app evaluations within a given round, meetings were held among the assessors to discuss any disagreements. For each app, a consensus decision on a final response for each assessment question was reached, which was passed on to the respective app developers. The information gathered during these discussions, together with the interrater reliability results, time and clarity ratings, and the feedback collected from developers, was used to amend and evolve the assessor handbook and developer guidance between rounds (and after the final round). Due to these iterative changes, the results are reported separately for each round.

### Data Analysis

Interrater reliability was determined for the agreement between the 2 assessment organizations based on their independent responses recorded before any discussion. For this purpose, Gwet AC1 values [31,32] were calculated for each assessment question in MATLAB (version 9.10.0.1710957 [R2021a]; The MathWorks, Inc). The Gwet AC1 was used as it has been shown to provide stable interrater reliability coefficients without being substantially affected by response prevalence or marginal probability (as is, eg, the case for the Cohen κ). In addition, it is suitable for designs that are not fully crossed over, such as the one used in this study, in which *different pairs* of raters evaluated the varying apps [33].

Gwet AC1 values can be interpreted as follows using Altman benchmark ranges [33]: <0.20="poor agreement," 0.21-0.40= "fair agreement," 0.41-0.60="moderate agreement," and >0.60="good agreement." The percentage of questions falling into each of these categories within each subsection of the CEN ISO/TS 82304-2, as well as across the entire assessment, was reported.

There were some instances in which assessors did not provide evaluation responses for individual questions, possibly because they were uncertain about which option to select (where coinciding clarity ratings were low). These instances were omitted from the analysis, as were a small number of cases in which an assessor responded with “not applicable” to a particular question even though this was not a valid response option. In addition, for 1 app in round 3, the evaluation responses from 1 assessment organization were not available due to technical issues. Therefore, this app was excluded from the interrater reliability analysis. Moreover, due to a miscommunication, 1 app in round 1 was evaluated by 3 assessment organizations instead of 2. As the assessors of one of these organizations were able to see the evaluation answers of another organization, the former’s responses were excluded from the interrater reliability (but not the time or clarity rating) analysis as a precaution to maintain full independence of responses. In subsequent rounds, additional measures were taken to ensure that access to other assessors’ responses was not possible.

For the time and clarity ratings collected from app developers and assessors, summary values were computed in MATLAB. Specifically, for each rating set (from a given rater for a particular app), the sum of the time ratings was calculated across all *questions* within a given section and across the entire assessment. The medians and IQRs of these values were reported as an indication of the central tendency and dispersion of the data *across rating sets.* A similar calculation was applied to the clarity ratings except that, for the latter, the average rather than the sum was computed across the questions within a given section before examining medians and IQRs across rating sets.

It should be noted that some assessors and developers provided time or clarity ratings for <75% of the questions within a particular section or across the entire assessment. These cases were excluded from the relevant section summary or the total assessment summary, respectively, as not enough data were available to arrive at a summary value that was likely to represent the rater’s time investment or perceived clarity (sample sizes are reported in the Results section). Where ratings were missing for <75% of questions, median imputation was applied using the available ratings for a given *question* before calculating the sum or average across all questions for a particular rating set. When calculating the summary values across the entire assessment (for cases in which <75% of all ratings were missing), median imputation was applied to *all* missing values, including to those within the sections that were excluded from the section summary (based on the aforementioned criterion). This was necessary for the calculation of the total amount of time spent on the assessment, for which time ratings were *summed* across *all* questions. The same methodology was applied to the clarity ratings for consistency.

### Ethical Considerations

Approval by an ethics or scientific research committee was deemed unnecessary according to Dutch national regulations, since the study was not subject to the Medical Research Involving Human Subjects Act, and according to the guidelines of the Central Committee on Research Involving Human Subjects. The study did not require ethics approval as no personal or sensitive data were collected and all activities performed by assessors and developers lay strictly within their professional competence and job responsibilities. Moreover, no identifiable information is disclosed about individual health apps in this paper.

## Results

### App Characteristics

Of the 24 apps included across the 3 assessment rounds, 8 (33%) were developed by micro-sized start-ups with <10 employees, 12 (50%) were developed by small businesses with 10 to 49 employees, 2 (8%) were developed by medium-sized companies with 50 to 249 employees, and 2 (8%) were developed by large organizations with ≥250 employees. Most companies (22/24, 92%) were based in Europe, that is, in the Netherlands (6/22, 27%), Belgium (4/22, 18%), and France (3/22, 14%), as well as in Bulgaria, Denmark, Finland, Greece, Italy, Portugal, Spain, Switzerland, and the United Kingdom (1/22, 5% each). The remaining companies (2/24, 8%) had headquarters in the United States.

The sample included both disease-specific and more general health or health system service support apps, with the former targeting a wide range of condition areas such as diabetes, asthma, cardiovascular disease, mental health, chronic pain, nicotine dependence, cognitive impairment, hepatitis C, and colorectal conditions. Most apps had more than one intended use, including health system service support (3/24, 12%), information provision (12/24, 50%), disease prevention (eg, through behavior change; 7/24, 29%), self-management (16/24, 67%), monitoring (15/24, 62%), treatment (4/24, 17%), communication with health care professionals (14/24, 58%), calculation of values that are likely to affect health care decisions (6/24, 25%), and research support (3/24, 12%). Overall, 46% (11/24) of the apps were classified as medical devices under the MDR.

### Interrater Reliability Between Assessment Organizations

For round 1, a median Gwet AC1 value of 0.54 (IQR 0.14-1) was observed across all 69 CEN ISO/TS 82304-2 assessment questions included in the analysis, indicating moderate overall agreement between assessment organizations. Breaking this finding down further revealed that the interrater reliability was poor (<0.20) for 32% (22/69) of the questions, fair (0.21-0.40) for 4% (3/69) of the questions, moderate (0.41-0.60) for 29% (20/69) of the questions, and good (>0.60) for 35% (24/69) of the questions.

In round 2, the overall interrater reliability between assessment organizations was good, with a median Gwet AC1 value of 0.63 (IQR 0.48-0.84). Agreement was poor (<0.20) for 7% (5/69) of the questions, fair (0.21-0.40) for 4% (3/69) of the questions, moderate (0.41-0.60) for 26% (18/69) of the questions, and good (>0.60) for 62% (43/69) of the questions.

In round 3, a median Gwet AC1 value of 0.67 (IQR 0.42-0.85) indicated good overall agreement between organizations. Interrater reliability was poor (<0.20) for 6% (4/69) of the questions, fair (0.21-0.40) for 14% (10/69) of the questions, moderate (0.41-0.60) for 20% (14/69) of the questions, and good (>0.60) for 59% (41/69) of the questions.

**Figure 1:**
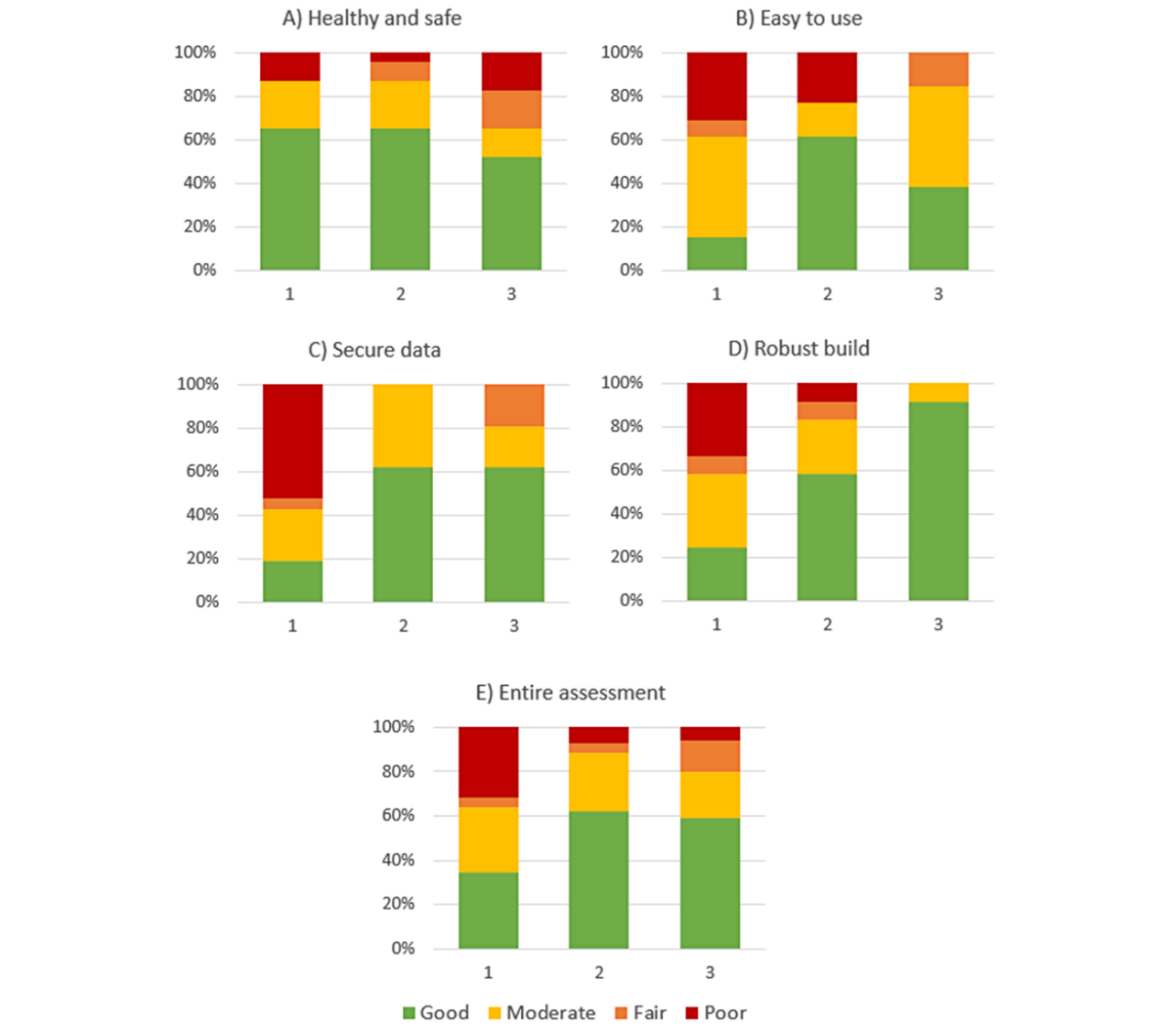
Percentage of questions falling into each interrater reliability category for the agreement between assessment organizations (Gwet AC1≤0.20="poor agreement," 0.21-0.40="fair agreement," 0.41-0.60="moderate agreement," and >0.6="good agreement"). Results are shown for the following assessment sections (A) "Healthy and safe" (23 questions), (B) "Easy to use" (13 questions), (C) "Secure data" (21 questions), and (D) "Robust build" (12 questions), as well as for (E) the Entire assessment (69 questions). Note that the "Product information" section only contained free-text and multiple-response options and, therefore, was not included in the interrater reliability analysis.

A visual representation of the interrater reliability findings by CEN ISO/TS 82304-2 subsection can be found in Figure 1.

### Time and Clarity Ratings

#### Assessor Ratings

The median evaluation time across all CEN ISO/TS 82304-2 questions reported by the assessors was 10 hours and 34 minutes (IQR 9.5-13.1 hours) in round 1 (n=5 rating sets), 8 hours and 33 minutes (IQR 4.9-9.0 hours) in round 2 (n=11 rating sets), and 8 hours and 46 minutes (IQR 7.1-11.0 hours) in round 3 (n=19 rating sets). The amount of time spent on each CEN ISO/TS 82304-2 subsection is presented in Table 1. It should be noted that these ratings include only the time it took to arrive at a response to the assessment questions based on the submitted evidence but not the time dedicated to other activities, such as meetings.

The median assessor handbook clarity rating across all questions was 1.46 (IQR 1.44-1.65) in round 1 (n=6 rating sets), 1.72 (IQR 1.53-1.88) in round 2 (n=10 rating sets), and 1.78 (IQR 1.71-1.89) in round 3 (n=12 rating sets), where 0=“very unclear”, 1=“somewhat unclear”, and 2=“completely clear”. Clarity ratings broken down by subsection can be found in Table 2.

**Table 1:** Evaluation time reported by the assessors across rounds for each subsection of the CEN ISO/TS 82304-2 assessment.

Section and round			Number of rating sets		Evaluation time (hours), median (IQR)
**Product information**					
	1	5		0.6 (0.5-0.7)	
	2	11		0.5 (0.2-0.5)	
	3	19		0.4 (0.3-0.7)	
**Healthy and safe**					
	1	5		3.6 (3.2-4.3)	
	2	11		3.2 (1.7-3.4)	
	3	19		3.1 (2.1-3.7)	
**Easy to use**					
	1	5		1.9 (1.7-3.1)	
	2	11		1.5 (0.8-1.8)	
	3	19		1.8 (1.3-2.3)	
**Secure data**					
	1	5		2.9 (2.6-3.4)	
	2	11		2.0 (1.2-2.3)	
	3	19		2.6 (1.0-3.3)	
**Robust build**					
	1	4		1.5 (1.2-1.9)	
	2	10		1.1 (0.7-1.4)	
	3	18		1.1 (0.1-1.7)	

**Table 2:** Handbook clarity ratings reported by the assessors across evaluation rounds for each subsection of the CEN ISO/TS 82304-2 assessment.

Section and round		Number of rating sets	Assessor clarity rating (0=very unclear, 1=somewhat unclear, and 2=completely clear), median (IQR)
**Product information**			
	1	6	2 (2-2)
	2	9	2 (2-2)
	3	14	2 (2-2)
**Healthy and safe**			
	1	6	1.54 (1.5-1.57)
	2	10	1.84 (1.29-1.96)
	3	12	1.88 (1.7-1.97)
**Easy to use**			
	1	6	1.54 (1.31-1.54)
	2	10	1.62 (0.62-1.77)
	3	9	1.54 (1.17-1.94)
**Secure data**			
	1	6	1.5 (1.24-1.67)
	2	10	1.9 (1.62-2)
	3	8	1.74 (1.52-2)
**Robust build**			
	1	6	1.21 (1.08-1.83)
	2	10	2 (1.67-2)
	3	13	1.92 (1.67-2)

#### App Developer Ratings

The median time spent by app developers on preparing the assessment information using the evolving guidance was 2 hours in round 1 (n=1 rating set), 7 hours and 3 minutes (IQR 3.8-8.8 hours) in round 2 (n=5 rating sets), and 8 hours and 59 minutes (IQR 5.7-27.1 hours) in round 3 (n=10 rating sets). The information collation time for each CEN ISO/TS 82304-2 subsection can be found in Table 3.

The median guidance clarity rating indicated by app developers across all questions was 1.84 (IQR 1.69-1.99) in round 1 (n=2 rating sets), 1.80 (IQR 1.74-1.87) in round 2 (n=5 rating sets), and 1.73 (IQR 1.64-1.90) in round 3 (n=12 rating sets). Clarity ratings broken down by subsection are presented in Table 4.

**Table 3:** Information collation time reported by app developers across rounds for each subsection of the CEN ISO/TS 82304-2 assessment.

Section and round		Number of rating sets	Information collation time (hours), median (IQR)
**Product information**			
	1	1	0.06 (—^a^)
	2	5	0.22 (0.16-0.31)
	3	10	0.48 (0.27-0.82)
**Healthy and safe**			
	1	1	0.5 (—)
	2	5	2.14 (1.32-3.4)
	3	10	4.11 (2.63-7.65)
**Easy to use**			
	1	1	0.26 (—)
	2	5	1.53 (0.8-2.22)
	3	8	1.8 (0.96-3.75)
**Secure data**			
	1	1	0.92 (—)
	2	5	1.32 (1.1-2.13)
	3	10	1.56 (0.99-8.5)
**Robust build**			
	1	1	0.28 (—)
	2	5	0.77 (0.38-1.46)
	3	9	0.9 (0.63-3.45)

^a^Not applicable.

**Table 4:** Guidance clarity ratings reported by app developers across evaluation rounds for each subsection of the CEN ISO/TS 82304-2 assessment.

Section and round		Number of rating sets	Developer clarity ratings (0=very unclear, 1=somewhat unclear, and 2=completely clear), median (IQR)
**Product information**			
	1	3	2 (1.36-2)
	2	5	2 (1.93-2)
	3	13	2 (1.96-2)
**Healthy and safe**			
	1	2	1.82 (1.64-2)
	2	5	1.93 (1.56-1.97)
	3	12	1.87 (1.65-1.92)
**Easy to use**			
	1	2	1.81 (1.62-2)
	2	5	1.92 (1.65-2)
	3	12	1.71 (1.65-1.88)
**Secure data**			
	1	2	1.98 (1.95-2)
	2	5	1.95 (1.6-1.96)
	3	12	1.81 (1.67-1.95)
**Robust build**			
	1	2	1.58 (1.25-1.92)
	2	5	1.83 (1.71-1.94)
	3	12	1.67 (1.33-1.88)

### Feedback From App Developers

All 24 app developers took part in individual follow-up interviews, which were conducted vial video calls after the developers had submitted their assessment information but before they received the evaluation responses from the assessors. The interviews were led by MK and took between 25 and 75 minutes. On the developer side, between 1 and 5 team members attended the interviews, which in all cases included individuals who had been involved in collating the assessment information and in 92% (22/24) of the cases included the person leading this process.

When asked what improvements they would recommend for the draft version of the assessment guidance and the evolving evaluation process, developers provided several suggestions. First, in rounds 1 and 2, it was noted that some questions were mainly applicable to medical apps, causing uncertainties about whether and, if so, how they could be answered for wellness apps. It was suggested to include stratification points in the assessment to determine which questions need to be answered for which type of app (which is part of the CEN ISO/TS 82304-2 framework but had not yet been applied in the draft guidance). Second, uncertainties were reported regarding what evidence was required for some of the questions. It was indicated that the provision of example answers and document templates, as well as the opportunity to interact with assessors as needed (which was always intended for the real-world assessment implementation), would be helpful to clarify requirements. Third, it was noted, especially by companies that outsourced their software development, that it would be useful to receive more explicit information on which expertise (eg, clinical, data privacy, cybersecurity, and software development) was needed to answer each individual question to allow for a more efficient distribution of the questions across the team and subcontractors. Fourth, developers asked for guidance on how to efficiently integrate CEN ISO/TS 82304-2 into their internal processes and strategy alongside other required assessments. In this context, it was noted that there is overlap between MDR and CEN ISO/TS 82304-2 requirements (which was deliberate and necessary to cover relevant quality criteria for apps that are not classified as medical devices or, given their class-I classification, not assessed by notified bodies). It was suggested to allow for the reuse of documentation or the automatic passing of certain CEN ISO/TS 82304-2 questions if an app had already been assessed by a notified body (which was indeed always intended but requires further consideration for effective implementation).

Developers were further asked which, if any, benefits they thought the CEN ISO/TS 82304-2 assessment and the associated quality label could provide for their company. In response to this question, 67% (16/24) of the developers indicated that they expected the quality label to be an asset for marketing purposes, in addition to creating confidence and trust among users, payers, and health care providers. A further 25% (6/24) of the developers primarily mentioned receiving an independent quality certification as a benefit in and of itself. Moreover, 21% (5/24) of the developers saw the assessment process as a valuable learning experience for the company, offering guidance on how to measure and improve the quality of their app, with 1 company reporting that they created an internal quality management system as a result of going through the assessment process.

Finally, when asked whether, knowing what they did now about the time and effort that was required, they would still have chosen to submit their app for CEN ISO/TS 82304-2 assessment, 88% (21/24) of the developers replied in the affirmative, with 8% (2/24) being unsure and 4% (1/24) indicating that they would not have submitted their app.

## Discussion

### Principal Findings

This study aimed to develop and evolve the CEN ISO/TS 82304-2 assessment handbook and developer guidance by testing them across a diverse range of apps and assessment organizations from different countries. Time spent on information collation or evaluation, instruction clarity, and assessment consistency were examined to inform the next steps toward establishing a robust certification process. To our knowledge, this is the first cross-national, cross-stakeholder collaboration in the field of health app assessment of this extent, diversity, and rigor.

This study revealed that the interrater reliability between assessment organizations improved from round 1 to round 2 for all assessment sections, likely reflecting both an initial practice effect and clarifications made to the assessor handbook. Between rounds 2 and 3, interrater agreement improved or stayed relatively stable for the “Robust build” and “Secure data” sections, respectively. However, unexpectedly, the “Healthy and safe” and “Easy to use” sections demonstrated somewhat lower interrater reliability in round 3 than in round 2 (which likely also drove the very slight decrease in interrater reliability observed across the entire assessment between these rounds). The evolving rigor of the handbook between rounds likely contributed to this finding, which may have affected some sections more than others depending on the changes that were made. Moreover, differences in app characteristics may have also played a role. For instance, in round 2, only 29% (2/7) of the evaluated apps were classified as medical devices, whereas in round 3, this classification applied to 43% (6/14) of the apps. The greater complexity of medical device app functionalities may have made the latter more difficult to evaluate in some (but not all) domains compared to apps that are not medical devices. In the future, to the extent possible, the assessment outcomes of notified bodies will be used to inform those CEN ISO/TS 82304-2 quality requirements that overlap with the MDR for medical device apps, which will increase the consistency of the latter’s assessment. In addition, the improvements discussed in the Learnings and Planned Improvements to the Assessment section aim to ensure consistent evaluation across all assessment sections and app types. Still, even with the round-3 draft version of the assessment handbook, 59% (41/69) of the questions across the entire assessment demonstrated good interrater reliability, with another 20% (14/69) of the questions showing moderate agreement. These results are promising, indicating that, even at this early stage, the CEN ISO/TS 82304-2 assessment already results in largely consistent outcomes across organizations located in different countries. The implications of this finding are discussed further in the Envisioned Impact of the CEN ISO/TS 82304-2 Assessment section.

The median handbook clarity ratings reported by assessors increased steadily across rounds, as was expected due to increasing familiarity with the handbook and the improvements made to the latter between rounds based on the collected data. Somewhat surprisingly, the median guidance clarity ratings collected from app developers decreased across rounds. This may have been an unintended consequence of the iterative changes (increasing rigor) that were made to the guidance. For instance, in round 3, the subrequirements that were used in the assessor handbook were added to the developer guidance with the aim of clarifying evidence needs and assessor expectations. However, this may inadvertently have caused uncertainty among developers about how to answer the associated assessment questions, with seemingly only a minority of developers having taken the individual subrequirements into account. Alternatively, especially given the small sample size, the observed differences across rounds may have been a result of variations in the included apps and development companies, which were likely to affect how easily certain assessment questions could be answered. In either case, clarification of the written guidance, giving developers the opportunity to contact the assessment organizations with clarification questions, and other improvements discussed in the Learnings and Planned Improvements to the Assessment section will likely help enhance developers’ understanding of the guidance in the future.

With regard to the time spent on the assessment process, in round 3, developers reported taking a median of 8 hours and 59 minutes (IQR 5.7-27.1 hours) to gather the assessment information, and assessors indicated spending a median of 8 hours and 46 minutes (IQR 7.1-1.0 hours) evaluating the apps. While this is a substantial time investment, it reflects the rigor of the CEN ISO/TS 82304-2 assessment, which is intended to ensure that different stakeholder groups internationally are provided with sufficient information to support their health app–related decision-making, significantly reducing the need for further evidence gathering and avoiding similar evaluations. Importantly, it should be noted that, despite the effort involved, 88% (21/24) of the app developers reported that they would submit their app for CEN ISO/TS 82304-2 assessment again, indicating that the perceived benefits of the assessment outweigh the time cost. In addition, several steps are being considered to reduce the required time for both developers and assessors in the future implementation of the assessment, as discussed in the Learnings and Planned Improvements to the Assessment section.

All in all, this study provided promising results regarding the interrater reliability between assessors at this early stage of development and testing, as well as valuable insights to support the future implementation of the CEN ISO/TS 82304-2 assessment. In the following sections, it is discussed how these results compare to previous findings; which improvements were made based on this study; what next steps are planned for the refinement of the CEN ISO/TS 82304-2 assessment handbook, developer guidance, and process; and what benefits the widespread use of the assessment is expected to provide. Strengths and limitations of the study are also considered.

### Comparison to Previous Health App Assessment Interrater Reliability Studies

Previous studies that have examined the interrater reliability of health app assessments have yielded mixed results. For several assessments, <15% of the questions demonstrated good agreement between assessors (eg, Fleiss κ>0.60 or Krippendorff α>0.667) [34-36]. Our results compare favorably to these findings, with approximately 60% of CEN ISO/TS 82304-2 questions in rounds 2 and 3 (43/69, 62%, and 41/69, 59%, respectively) showing good assessor agreement (Gwet AC1>0.60), already at this early stage of development. However, it should be noted that higher interrater reliability has been reported for several health app assessments, such as outstanding agreement (κ>0.80) for 86% of the Enlight questions [37], substantial or good agreement (κ>0.60) for 79% of the questions within the American Psychiatric Association’s app evaluation framework [38], and satisfactory intraclass correlations (>0.75) for all sections of the Mobile App Rating Scale [39].

In this context, the complexity of the assessments should be considered. The aforementioned frameworks are less comprehensive in scope, rely only on publicly available information (mostly the app itself), and include less complex and often more descriptive questions than those in the CEN ISO/TS 82304‑2 assessment. Therefore, it is unsurprising that good interrater reliability could be achieved relatively easily for these frameworks. In contrast, the in-depth evaluation of clinical and technical aspects provided as part of the CEN ISO/TS 82304-2 assessment requires expert assessment knowledge and careful examination of elaborate documentation submitted by developers. Consequently, it is to be expected that repeated testing and iterative improvement are required to achieve a clear handbook and good assessor agreement for all CEN ISO/TS 82304-2 questions. In line with this, a recent qualitative study found that, even for the MDR, there are significant variations in the interpretation of the included quality requirements both within and between notified bodies, which can result in inconsistent assessment outcomes [40]. This highlights the difficulty of achieving consistency for more complex digital health technology quality assessments. In this context, it is worth noting that, unlike some other quality or efficiency assessments (eg, those carried out for the EU energy label), digital health technology assessments involve evaluations carried out by assessors rather than relying only on automated measurements (eg, of energy consumption). This introduces variability, which needs to be minimized to ensure that national authorities, citizens, and health care professionals can trust that health apps are evaluated consistently. The refinements discussed in the Learnings and Planned Improvements to the Assessment section aim to further improve the CEN ISO/TS 82304-2 assessment consistency. This includes amendment of the handbook and guidance as well as considerations related to the expertise, training, and support from subject matter experts and automated tooling that can assist assessors in achieving consistent ratings.

With regard to the time required for app evaluation, the few previous interrater reliability studies that have examined this measure have reported assessment times ranging from 15 minutes to 1 hour [34,41]. These values are considerably lower than those observed in this study, with a median evaluation time of 8 hours and 46 minutes (IQR 7.1-11.0 hours) reported in round 3. Again, this difference is likely due to the reliance only on publicly available information and the lower complexity of the former assessments compared to that of the CEN ISO/TS 82304-2. Pilot studies more similar to this one, as part of which information was requested from health app developers, have reported app evaluation times more comparable to those in our study. Specifically, one study observed assessment times of 10 to 30 work hours per app (including writing the recommendation, meetings, and other communications [42]), and another study (including 2 apps) reported indicative app evaluation times of up to 6 hours (if uncertainties were encountered [43]). The time information gathered during this study will be used to inform developers of how long it is expected to take to collate the assessment information (developer workload), determine assessment pricing, and guide scalability efforts (assessor workload).

### Learnings and Planned Improvements to the Assessment

On the basis of the information collected during this study and complementary studies conducted as part of the initiative that this study is a part of, improvements were made to further increase the consistency, clarity, efficiency, and value of the assessment. Several of these learnings may also be applicable to other health app evaluation initiatives.

First, to increase consistency, the handbook was further evolved to include separate sections with standardized, simplified wording. These sections provide additional guidance as to (1) the documentation needed from app developers; (2) which subrequirements the assessors need to evaluate with the provided documentation and, if applicable, with information from the app itself; and (3) which subrequirements need to be met for the assessment question to be answered with a “yes.” In addition, it is being explored whether automation can be introduced to evaluate or support the evaluation of some requirements, such as testing accessibility features, examining data flow, and scanning privacy policies for relevant content. In addition to improving consistency, automation can help reduce the required assessment time and may enable the evaluation of additional quality aspects that cannot be examined manually. Moreover, in the future, the outcomes of trusted existing assessments, in particular those provided by notified bodies as part of their legal task, will, to the extent possible, be used to address the related CEN ISO/TS 82304-2 requirements, which is expected to improve consistency as well as avoid duplication of effort. Finally, consideration is being given to how best to train new assessors (eg, using practice evaluations on already assessed apps) to ensure that they demonstrate a high level of consistency before starting to conduct “real” app assessments.

Second, to increase clarity and efficiency, a fit-for-purpose platform, including subsection headings, stratification, and documentation templates, is intended to be developed to collect assessment information from developers. Subsection headings will make the expertise needed to answer different questions (eg, medical, data privacy, or cybersecurity knowledge) more apparent. This can help developers distribute the assessment questions more effectively across the company and potential suppliers, asking relevant experts to answer the questions most suited to their expertise, thereby saving time in collating the assessment information. Stratification, carried out automatically via the platform, will more efficiently determine which assessment questions need to be answered based on the intended use and characteristics of the app, such as whether it provides health information, has already been assessed by a notified body, processes personal data, or incorporates application programming interfaces. Document templates, which interviewed developers suggested would be helpful, will further clarify the evidence requirements and allow developers to identify more quickly and effectively which information they need to provide. This, in turn, will ensure that assessors receive all and only the information needed for the evaluation in a standard format, speeding up the evaluation process and making it more robust.

Efficiency is also expected to be increased by giving developers and assessors the opportunity to interact as part of the assessment process (as was always intended). This was not the case during the study to enable the examination of developers’ understanding of the guidance in the absence of outside advice and to ensure that assessors’ evaluation responses were independent and based on the same information. Due to the lack of interaction during this study, developers were occasionally uncertain about how to respond to particular questions or what documentation to provide, and in some instances, assessors spent considerable amounts of time searching for relevant information, including within the apps. In the real-world implementation of the assessment, developers will have the chance to ask clarification questions, and assessors will be able to contact developers to request additional information if needed. It is expected that such interactions will reduce uncertainty and expedite the evaluation process. Future consideration will additionally be given to how to efficiently handle reassessments where changes to an app have been made that affect the responses to individual quality requirement questions and how to guide developers in efficiently integrating CEN ISO/TS 82304-2 into their internal processes and strategy.

Third, to increase the value and uptake potential of the CEN ISO/TS 82304-2 assessment, steps were taken to evolve and align it more specifically with the European context, where the initial implementation efforts are intended to take place. This included incorporation of findings of a comparative analysis with (mostly) European health technology assessment frameworks, EU-level legislation and values, international and European standards, advice from subject matter experts, literature on health app quality, and information needs reported by (mostly) European stakeholders for health app–related decision-making. Consultation of related European authorities such as the Medical Device Coordination Group, the European Data Protection Supervisor, and the EU Agency for Cybersecurity is proposed for the future to obtain feedback and ensure sustained alignment. In addition, future work will aim to align the assessment with regulations and standards in other regions as well. Notably, it has been observed that many regulations are promulgated from the EU to other regions, where they become entrenched in relevant legal frameworks (which has been termed the “Brussels effect” [44]). Therefore, by aligning the handbook with EU regulations such as the MDR and General Data Protection Regulation, many (often less rigorous) legal requirements in other regions are likely already covered, and future efforts will likely only require relatively small additions (or removal) of requirements to align with local legislation. Once the assessment is taken up in different regions, a flag displayed at the top of the CEN ISO/TS 82304-2 quality label can indicate in which region the assessment was conducted (or recognized). This is similar to the EU energy label, which is used by many countries outside the EU with some, strong, or full alignment and displays the region or country flag at the top of the label [45].

Finally, it should be noted that the initial focus of the development and testing effort was on smaller organizations and start-ups, assuming that, if the assessment process was optimized for the latter, it would also be suitable for larger companies that tend to employ more subject matter experts. However, this study revealed that larger organizations may require somewhat different guidance from that required by small or medium-sized businesses due to differing organizational structures and interrelations between products and associated documentation. For instance, in large companies, a given health app may be a supportive tool for a medical intervention that is not delivered through the app itself, and documentation for these connected products may not be easily separable. Relatedly, certain policies in place at large companies, such as data privacy provisions, may be applicable to many offered products rather than being health app specific. In addition, in some cases, larger companies may have (recently) acquired smaller app development organizations, with only some but not all documentation already having been updated to align with the larger company’s standard procedures. Such scenarios may cause uncertainties within large companies regarding what documentation is suitable and relevant for the app-specific CEN ISO/TS 82304-2 assessment if the evaluation guidance is mainly aligned with the needs of smaller organizations. As only 2 large companies were involved in this pilot study, additional testing is needed and planned to calibrate the assessment guidance for use in large organizations.

### Envisioned Impact of the CEN ISO/TS 82304-2 Assessment

As mentioned in the Introduction section, the need for a health app quality certification beyond that provided under the MDR seems to be widely recognized [15,19,20], but current health app assessment initiatives, including national programs, are struggling to achieve efficient, appealing, and scalable assessment implementation. This may partly be due to the use of centralized national approaches to app evaluation, which can create bottlenecks [29], as well as due to insufficient incentives for developers. It has been proposed that international collaboration and the use of distributed evaluation bodies is needed to address these issues [29]. As such, the envisioned establishment of the CEN ISO/TS 82304-2 assessment as an efficient, effective, consistent, and scalable health app quality certification scheme that is delivered through a network of assessment organizations and is recognized across Europe and beyond, requiring only limited context-specific requirements to be added, would be a major step forward.

It is expected that the widespread availability of the CEN ISO/TS 82304-2 quality label will minimize risk and build trust among health care professionals and the public, thereby increasing the adoption of high-quality health apps. In line with this expectation, our recent research has shown that health care professionals’ willingness to recommend health apps and users’ intention to download them is positively influenced by the CEN ISO/TS 82304-2 quality label ([46]; Adriaanse, unpublished data).

In addition, the cross-national recognition of the CEN ISO/TS 82304-2 assessment would help address several issues associated with the current fragmented assessment landscape in which numerous organizations apply differing assessment frameworks for overlapping quality aspects [19,20]. This fragmentation leads to duplicated work both for health systems and app developers if a given app needs to be submitted to several full assessments to evidence its quality to different markets and varying types of organizations. In this regard, it is specifically problematic that the quality criteria used can differ widely across frameworks, with a recent review finding that, among 12 health system- and government-led digital health quality assessments, the criteria overlap in some domains was <50% [20]. Such inconsistencies in quality standards can cause additional work (if varying evidence needs to be prepared and submitted) and confusion among both developers and users, potentially jeopardizing trust in quality evaluations if the same health app may fail one assessment but pass another. In addition, the need for an app to undergo several assessments to enter various markets may lead to inequities. While developers may regard it as worthwhile to invest time and money to receive quality certifications in countries with large populations, this is less likely to be the case for smaller markets, for which the cost-benefit ratio of undergoing an additional assessment and addressing local evidence needs, such as a study conducted within the related country, may not be appealing. This may lead to a situation in which some high-quality apps are not available in certain regions or languages.

The CEN ISO/TS 82304-2 assessment is intended to address these issues, avoiding duplication of work, preventing inequities from arising, and building trust by serving as a “gold standard” assessment that is recognized across a wide range of countries and types of organizations. A recent paper with the European Society of Cardiology [47] and preliminary results from a comparative analysis with European health technology assessment frameworks (Hoogendoorn, unpublished data) indicate that the CEN ISO/TS 82304-2 assessment covers the vast majority of criteria that are regarded as important to evidence health app quality. By primarily relying on the CEN ISO/TS 82304-2 quality label and only carrying out additional, context-specific evaluations if the label indicates that a given app is of acceptable quality, health systems and other stakeholders can obtain substantial time and resource savings during health app–related decision-making and create streamlined processes for developers.

### Strengths and Limitations

This study took an innovative and ambitious approach to health app quality assessment by engaging organizations from 5 countries in the evaluation process and examining their interrater reliability. In addition, 24 health app development companies were involved in this study, and their feedback was sought. To our knowledge, this is the first such collaboration, which lays the foundation for the consistency and scaling of the CEN ISO/TS 82304-2 assessment across Europe and beyond.

However, some limitations should be noted. First, the sample size of included apps was small (given that each app was rated by only 2 assessment organizations), limiting the generalizability of the findings. This was partly due to the funding and time constraints of the project, as well as due to developer dropouts, especially in the first assessment round. Given the small sample involved in this pilot study and the evolving nature of the guidance and handbook throughout the study and beyond, future studies with a larger sample and using the operational version of the guidance and handbook are required to provide conclusive evidence for the interrater reliability of the CEN ISO/TS 82304-2 assessment.

Second, as mentioned previously, relatively high developer dropout rates were observed, which may have distorted the findings if developers who found the guidance more difficult to understand were more likely to drop out. However, this is unlikely to have been the case as many of the dropouts occurred before developers had even received the guidance, and across all rounds, only 3 developers dropped out after they had started to collate the assessment information. In this regard, it should also be emphasized that developers were not provided with a quality label as part of this study given that the aim was to develop and refine the CEN ISO/TS 82304-2 assessment handbook and guidance before arriving at an operational version that is suitable for use in quality certification. This likely contributed to the dropouts given that, as indicated during the developer interviews, receiving a quality label to build trust and support marketing efforts is the main benefit of the assessment for developers. It is expected that dropouts will be less common in the future implementation of the assessment once a quality label is issued and developers actively seek out the assessment of their apps to gain the benefits of the label.

Third, given that this study aimed to approximate real-world conditions with a focus on app, developer, and assessor diversity, some aspects were not as tightly controlled as they could have been, such as the application of eligibility criteria or the number of individual assessors involved in the evaluation of a given app across organizations. This was deemed necessary to allow for the detection and solution of potential issues that may arise during the real-life assessment process. Moreover, this approach ensured that the gathered consistency, time, and clarity data were representative of what would be expected in the real world (with the draft handbook and guidance) without being (potentially positively) distorted by artificially imposed conditions that will not be present once the assessment is rolled out.

### Conclusions

This study involved an unprecedented collaboration across organizations in 5 countries to develop, test, and evolve the CEN ISO/TS 82304-2 health app quality assessment handbook and guidance. Promising findings were obtained, especially given the early stage of development, with regard to the interrater reliability between assessors, and valuable information was gathered to support the future evolution and implementation of the assessment. These are important steps toward establishing the CEN ISO/TS 82304-2 assessment as a commonly recognized health app quality evaluation across Europe and beyond. The widespread use of the CEN ISO/TS 82304-2 assessment across countries is expected to have a substantial positive impact, avoiding duplication of work, preventing inequities by facilitating access to smaller markets for developers, enhancing the quality of apps, and building trust in health apps among health care professionals and the public. This can ultimately help increase the adoption of high-quality health apps that have the potential to improve health outcomes and health care system efficiency.

## Abbreviations

EU: European Union
MDR: Medical Device Regulation

## Data Availability

The datasets generated or analyzed during this study are available from the corresponding author on reasonable request.
